# The Double Standard of Aging for Men and Women: Evidence From Across Europe, 2006–2018

**DOI:** 10.1093/geroni/igaa057.1829

**Published:** 2020-12-16

**Authors:** Richard Settersten, Jack Day, Gunhild Hagestad

**Affiliations:** 1 Oregon State University, Corvallis, Oregon, United States; 2 SUNY-Oneonta, Oneonta, New York, United States; 3 Agder University & Northwestern University, Kristiansand, Vest-Agder, Norway

## Abstract

Is there a “double standard” (i.e., a harsher judgment) in the perceived ages at which women and men reach old age, and have these judgments changed over time? We use European Social Survey data from 23 countries in 2006 and newly released data from 16 of those countries in 2018. In both 2006 and 2018, men typically assign women substantially earlier ages than women themselves do. In some places, however, men also give themselves lower ages than women give them. With respect to when women become old, the differential views of men and women are persistent. So is the fact that women differentiate less between the sexes¬–though men differentiate less in 2018 relative to 2006. We use multilevel modeling to examine variation explained by both individual characteristics and country indicators of demographic and policy contexts. Findings underscore the significance of the double standard in cultural constructions of aging.

